# Lower Obesity Rate during Residence at High Altitude among a Military Population with Frequent Migration: A Quasi Experimental Model for Investigating Spatial Causation

**DOI:** 10.1371/journal.pone.0093493

**Published:** 2014-04-16

**Authors:** Jameson D. Voss, David B. Allison, Bryant J. Webber, Jean L. Otto, Leslie L. Clark

**Affiliations:** 1 Epidemiology Consult Division, US Air Force School of Aerospace Medicine, Wright Patterson Air Force Base, Ohio, United States of America; 2 Department of Preventive Medicine, Uniformed Services University, Bethesda, Maryland, United States of America; 3 Department of Biostatistics, University of Alabama at Birmingham, Birmingham, Alabama, United States of America; 4 Nutrition and Obesity Research Center, University of Alabama at Birmingham, Birmingham, Alabama, United States of America; 5 Trainee Health Surveillance, Joint Base San Antonio – Lackland, Lackland, Texas, United States of America; 6 Armed Forces Health Surveillance Center, Silver Spring, Maryland, United States of America; 7 Henry M. Jackson Foundation for the Advancement of Military Medicine, Bethesda, Maryland, United States of America; 8 General Dynamics Information Technology, Fairfax, Virginia, United States of America; Geisel School of Medicine at Dartmouth College, United States of America

## Abstract

We sought to evaluate whether residence at high altitude is associated with the development of obesity among those at increased risk of becoming obese. Obesity, a leading global health priority, is often refractory to care. A potentially novel intervention is hypoxia, which has demonstrated positive long-term metabolic effects in rats. Whether or not high altitude residence confers benefit in humans, however, remains unknown. Using a quasi-experimental, retrospective study design, we observed all outpatient medical encounters for overweight active component enlisted service members in the U.S. Army or Air Force from January 2006 to December 2012 who were stationed in the United States. We compared high altitude (>1.96 kilometers above sea level) duty assignment with low altitude (<0.98 kilometers). The outcome of interest was obesity related ICD-9 codes (278.00-01, V85.3x-V85.54) by Cox regression. We found service members had a lower hazard ratio (HR) of incident obesity diagnosis if stationed at high altitude as compared to low altitude (HR 0.59, 95% confidence interval [CI] 0.54–0.65; p<0.001). Using geographic distribution of obesity prevalence among civilians throughout the U.S. as a covariate (as measured by the Centers for Disease Control and Prevention and the REGARDS study) also predicted obesity onset among service members. In conclusion, high altitude residence predicts lower rates of new obesity diagnoses among overweight service members in the U.S. Army and Air Force. Future studies should assign exposure using randomization, clarify the mechanism(s) of this relationship, and assess the net balance of harms and benefits of high altitude on obesity prevention.

## Introduction

Obesity is a global health priority with medical, societal, financial, and security ramifications. [1], [2] It threatens the operational capacity of the U.S. military, both by its increasing prevalence among the present force [3] and its impact on the qualified applicant pool [4].

The burden of disease remains high despite implementation of several lifestyle-based public health interventions. Within the U.S. armed forces, despite universal access to free healthcare, high prevalence of physical activity [5] and regulations requiring a healthy body weight (Department of Defense Directive 1308.1), [6] excess weight diagnoses have increased markedly. [7] This is associated with decreased length of service [8], [9] and costs exceeding $1 billion annually. [10] Ultimately, more effective interventions are needed–both for this population and for individuals around the globe.

Hypoxia has been investigated as a hypophagic agent in rats since the 1960s [11]–[16] and has recently gained attention as a potential therapeutic agent in humans. [17], [18] Human interventional trials have demonstrated reduced appetite and body fat in hypoxic conditions, including high altitude travel, [19]–[31] although such trials have been of short duration. Recently, we documented an inverse, dose-response association between the altitude of one’s residence and obesity prevalence in the United States. [32]–[34] As compared to their counterparts residing in high altitude counties, individuals in low altitude counties had over 4 times the prevalence of obesity, after adjusting for diet, physical activity, smoking, demographic and other factors. Although this finding suggests a potential long-term metabolic benefit from hypoxia, the dataset lacked duration of residence and temporal sequence of exposure and outcome, and was thus limited by the potential of reverse causation. Other studies in Nepal, India, and Argentina have shown similar results [35]–[37].

This present study analyzes whether long-term residence at high altitude alters progression from overweight to obesity. As a large, relatively homogenous population with expansive and available demographic, occupational, geographic, and health records, the military population provides an unparalleled cohort for investigating this question. In addition, their migratory pattern derives from external orders (i.e., semi-random exposure assignment) rather than individual prerogative, representing a form of quasi-experiment [38].

## Materials and Methods

### Ethics Statement

This dataset was originally constructed for public health surveillance as approved by Reports and Request Review at the Armed Forces Health Surveillance Center. Analysis of the deidentified dataset for generalizable knowledge was exempted as non-human subjects research by the Air Force Research Lab Institutional Review Board at Wright-Patterson Air Force Base, OH.

### Methods

This quasi-experimental, retrospective study with a surveillance period of 1 January 2006 to 31 December 2012 included enlisted service members in the active component of the U.S. Army or Air Force with at least 2 years in service, an overweight (but not obese) enlistment body mass index (BMI≥25 & <30) and no prior diagnosis of obesity between the time of military enlistment to study entry. We were interested in evaluating an overweight population who would be at risk of progression to obesity during a single duty assignment.

**Figure 1:**
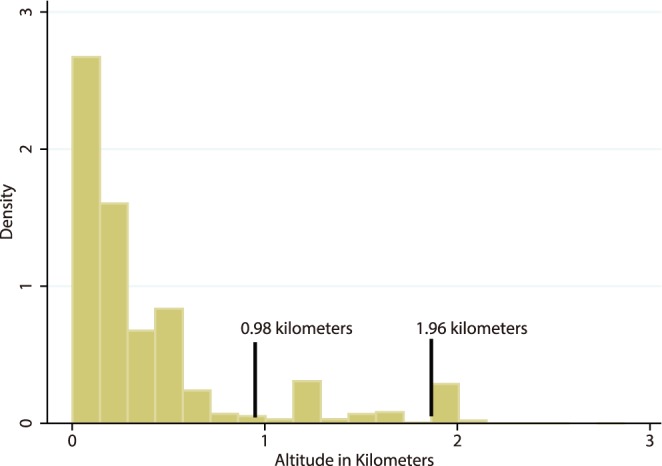
Histogram of Observed Density by Altitude. Lines represent altitude categorization based on natural break at 1.96

Once a military member met all inclusion criteria, the time they were observed was divided into “segments” of time representing the length of inclusion at a unique duty location assignment. The altitude of each military duty station was defined by the average altitude of the station’s 3-digit unit zip code from WorldClim shuttle radar topography maps [39] and derived with Geospatial Information System software (ArcGIS version 10.0). [40] Service members’ unit zip codes were obtained from demographic records in the Defense Medical Surveillance System (DMSS). [40] There were 28 observations from segments at a 3 digit zip code for which there was not an available altitude; these were included as a missing category. The other altitude categories were selected after data collection to create three equidistant intervals of 0.98 km, taking advantage of the natural break at 1.96 km (see Figure 1).

Additional demographic information was collected including age, self-reported race/ethnicity, sex, branch of military service, time in military service, occupation category, baseline BMI, and home of record (this refers to any location specified by the service member–which may indicate location of residence upon military entry or intended location of residence after completion of military service). Furthermore, to account for income disparities among military members based on duty location, the 2009 basic allowance for housing (BAH) averaged over each 3-digit zip (for the unmarried, lowest ranking pay-grade) was utilized as an additional covariate, based on publically available data. [41] A total of 85,098 segments (periods at a unique duty location) were excluded because the 3-digit zip reflected an Army (or Fleet) Post Office that was not located in a U.S. State (such as Europe or Guam) (n = 61,677), or was invalid (n = 23,421). One individual was excluded due to missing baseline BMI. If a model includes civilian obesity prevalence from NHANES, the New England census region is excluded due to insufficient observations from New England in this dataset [42].

The outcome of interest was incident clinical obesity, defined by at least one outpatient or inpatient medical encounter coded with the indicator *International Classification of Diseases, Ninth Revision, Clinical Modification* (ICD-9-CM) code of 278.00 or 278.01 (obesity) or V-code of V85.3x, V85.4, or V85.54 (BMI≥30) in any diagnostic position. A service member could be counted as an incident case only once.

All observations meeting inclusion criteria were analyzed using Cox regression with “time 0” defined as the time when an individual entered the study. Time 0 began in the middle of the surveillance period for service members who reached 2 years of active service during the period. For those with gaps in their personnel record lasting greater than 6 months, person-time was restarted at time 0 at the same location after the gap ended. Censoring occurred when a service member separated from military service or changed duty locations, or at the conclusion of the surveillance period. Service members with a change in duty location during the surveillance period contributed person-time at each location. To account for lack of independence when the same individual was observed at different duty stations, a variance estimator was used to cluster observations from the same individual (option “vce” clustering on the individual using Cox regression in Stata version 12). [43] Personnel records in DMSS only document “permanent” duty assignment and do not account for time spent away from that location (e.g., while on military leave, overseas deployments, and temporary duty assignments). Data analysis was performed using Stata v.11.0–12.1.

Geographic determinants other than altitude (e.g., rainfall, temperature, sunlight, obese social contacts, and cultural values) could vary between regions or states. Assuming no migration and steady state population weight (at the spatial resolution assessed), the cumulative (relative) effect of all of these geographic exposures should be revealed by the resultant obesity prevalence among civilians in these areas. The assumptions create limitations, and the spatial variation at this spatial resolution could be partially determined by altitude variation. Nonetheless, obesity prevalence provides a surrogate for other, unmeasurable geographic factors. Thus, civilian obesity rates were obtained from the Centers for Disease Control and Prevention’s (CDC) BRFSS for self-reported height and weight at the state level [44] and from the NHANES and REGARDS databases for measured height and weight based on the regional level as reported elsewhere. [42] These percentages were manually assigned to each zip code range based on publically available 3-digit zip codes for each state [45], [46].

Using obesity prevalence as a surrogate for unmeasured spatial determinants doesn’t account for time-dependent spatial features (i.e., features that exert a different effect based on length of residence). For example, smoking behavior varies spatially throughout the United States, [47] is socially transmitted, [48] and is associated with significantly more weight gain for recent than long-term quitters. [49] Because individual level smoking behaviors were not available, adjustments for state-level self-reported current smoking status (obtained from the 2011 BRFSS dataset) were used in robustness analyses [47].

To account for non-random aspects of the assignment process, such as personnel requests, which may influence the process differently by occupation, robustness analysis was performed. The seven occupational categories were stratified into separate analyses to assess the magnitude of the association across occupational substrata.

## Results

### Summary Characteristics

Summary demographics by exposure category are provided in Table 1. There were 98,009 individuals who contributed a median 3.2 years of exposure. The median length of each segment, reflecting unique duty locations, was 1.2 years of qualifying observation (time after diagnosis and time while members had less than two years of service not included).

**Table 1:** Demographic and Military Characteristics among Overweight Active Component Army and Air Force Service Members by Altitude, 2006–2012.

Baseline Covariates		Low Altitude (<0.98km)	HighAltitude (>1.96 km)	p-value*
Person-Years	Total (1000s)	300.4	16.1	–
Baseline Body Mass Index	Mean (kg/m^2^)	26.8	26.9	p<0.001
Age	Mean (years)	28.0	27.9	p<0.001
Time in Service	Mean (years)	8.0	7.8	p<0.001
Sex	Proportion Male	92.9	93.7	p<0.001
Service Branch	Proportion Army	64.5	76.6	p<0.001
Housing Allowance (E1)	Proportion below median	49.1	99.9	p<0.001
Occupation	Armor/transport	4.1	6.5	p<0.001
	Communication/Intelligence	26.2	26.0	
	Healthcare	7.6	5.9	
	Infantry/artillery/combat	4.6	6.2	
	Other	27.4	32.5	
	Repair/engineer	28.9	22.6	
	Pilot/aircrew	1.2	0.3	
Race/Ethnicity	Asian	3.6	3.5	p<0.001
	African American	19.3	12.8	
	Hispanic	12.1	12.6	
	American Indian	0.7	1.0	
	Other Race	1.1	0.9	
	Unknown	2.7	2.5	
	White	60.5	66.7	

*P-values based on χ square test of homogeneity for Sex, Service Branch, Housing Allowance, Occupation, and Race/Ethnicity and are based on unequal variance t-test for Age, Time in Service, and BMI. Statistical tests were not weighted for observation time.

There was a small but sizable portion of the population stationed at high altitude with 16,111 person-years observed. Demographic characteristics were generally similar by altitude category with black race and healthcare occupations being somewhat more common at low altitude than at high altitude. Military enlistment body mass indices (BMIs) were similar across strata of altitudes with high altitude having a slightly greater mean BMI. Covariates listed in Table 1 were included in the final model to correct for minor demographic differences.

### Primary Findings

**Figure 2:**
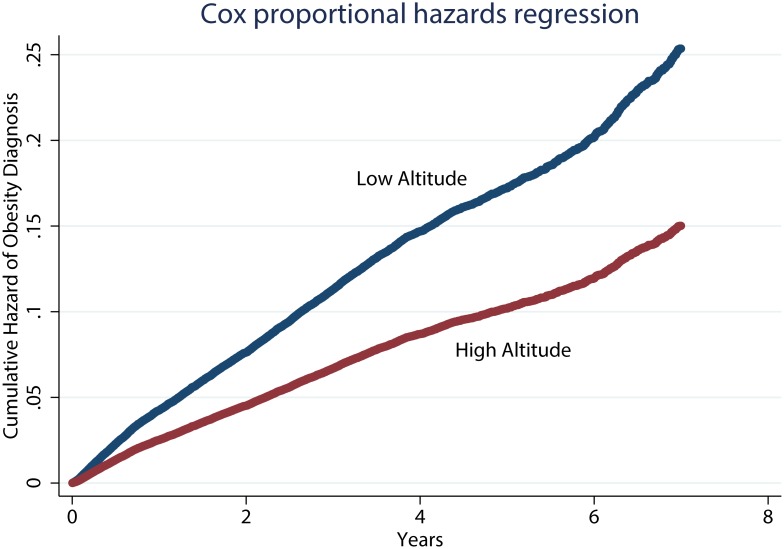
Cumulative Hazard Function. Cumulative Hazard of Obesity Diagnosis based on Cox Proportional Hazards Model Adjusted for Enlistment BMI, Sex, Race, Occupation, Time in Service, Branch of Service, Housing Allowance, and Age. The Red Curve is High Altitude and the Blue Curve is Low Altitude.

Service members stationed at high altitude had a 41% (95% confidence interval [CI] 35%–46%; p<0.001) lower hazard rate of obesity as compared to those stationed at low altitude, after controlling for enlistment BMI, branch of service, time in service, occupation, sex, race/ethnicity, age, and housing allowance (Table 2 and Figure 2). Unadjusted results were similar (41%; 95% CI 35%–47%; p<0.001). Relative to those in the healthcare career field, those in aircrew occupations had a 46% (95% CI 34%–56%) lower hazard rate of an obesity diagnosis.

**Table 2:** Hazard Ratios by Fully Adjusted Cox Model.

	Variables	Hazard Ratio (95% CI)
General	Air Force (vs. Army)	1.39 (1.33–1.44)
	Years in Service	1.03 (1.02–1.03)
	Age	0.99 (0.98–0.99)
	Enlistment BMI (−25)	1.32 (1.30–1.33)
	Sex (M vs. F)	0.51 (0.48–0.54)
	BAH (per $100)	0.98 (0.97–0.98)
Race/Ethnicity	Asian	Referent
	White	1.10 (1.00–1.21)
	Black	1.28 (1.16–1.41)
	Hispanic	1.20 (1.08–1.33)
	American Indian	1.21 (0.99–1.47)
	Other	1.00 (0.83–1.20)
	Unknown	1.16 (1.01–1.33)
Job Type	Armor/transport	0.99 (0.90–1.09)
	Communication/Intel	0.86 (0.80–0.91)
	Healthcare	Referent
	Infantry/artillery/combat	0.88 (0.80–0.97)
	Other	0.74 (0.69–0.79)
	Repair/engineer	0.91 (0.85–0.97)
	Aircrew	0.54 (0.44–0.66)
Altitude Category	Low Altitude	Referent
	Medium Altitude	0.95 (0.90–1.00)
	High Altitude	0.59 (0.54–0.65)
	Missing Altitude	0.96 (0.25–3.70)

### Robustness Analyses

**Figure 3:**
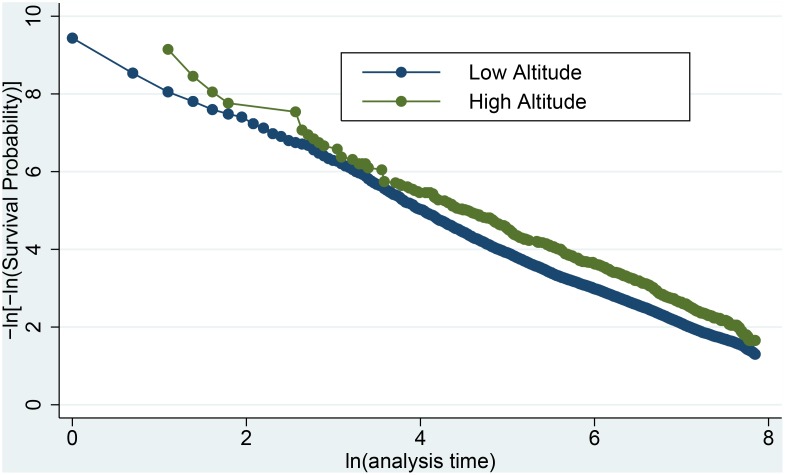
Log-Log Plot for Goodness of Fit with Proportional Hazards Assumption. The x-axis represents the natural log of analysis time in days.

Several analyses were performed to investigate the robustness of the findings. Model diagnostics demonstrated consistency of proportional hazard throughout the seven-year period (Figure 3). Those identifying Colorado as their “home of record” could have increased reason to request Colorado duty assignment. Controlling for Colorado “home of record,” however, altered the hazard ratio (HR) of high altitude only slightly from 0.59 to 0.60. Manually assigning altitude categories to the 28 observations with missing altitude did not alter the results (HR 0.59, 95% CI 0.54–0.65).

When dividing altitude into three equal intervals of 0.78 km based on the range of available altitudes (0–2.34 km) independent of natural breaks, those stationed at high altitude (>1.56 km) had lower hazard of obesity (HR 0.77; 95% CI 0.72–0.83) than those at low altitude in the fully adjusted model, but some individual duty locations above 1.56 km did not have a protective association. Alternatively, substituting altitude as a continuous variable, there was a 6% lower hazard of obesity (HR 0.94; 95% CI 0.91–0.96) per kilometer gained above sea level.

When included as a covariate, civilian obesity prevalence (as defined by Behavioral Risk Factor Surveillance System [BRFSS], National Health and Nutrition Examination Survey [NHANES], and Reasons for Geographic and Racial Differences in Stroke [REGARDS] datasets) was strongly related to military obesity incidence. After incorporating prevalence from all three civilian datasets in the fully adjusted model, high altitude duty assignment remained a protective association (HR 0.83; 95% CI 0.73–0.95) as compared to low altitude. This model predicted a 10% increase (HR 1.10; 95% CI 1.09–1.11) in obesity incidence among service members for every 1% increase in regional obesity prevalence as determined by REGARDS. The relationship between civilian obesity prevalence and hazard of obesity diagnosis remained consistent even when limiting the dataset to those stationed at low altitude (i.e., the unexposed) (HR 1.11; 95% CI 1.10–1.12). Further, all three independent data sources demonstrated an increased hazard of obesity diagnosis in areas with increased obesity prevalence (Table 3). Adjustment based only on actual measurements of height and weight (i.e., using NHANES or REGARDS) resulted in high altitude duty assignment having a non-significant protective association (NHANES: HR 0.94; 95% CI 0.84–1.07; and REGARDS: HR 0.91; 95% CI 0.82–1.02 – Table 3).

**Table 3:** Hazard of Obesity Incidence Based on Different Measures of Civilian Obesity.

		Hazard Ratio (95% CI)	
		Model 1*	Model 2†
REGARDS	REGARDS Obesity Prevalence	1.07 (1.07–1.08)	1.08 (1.07–1.09)
	High Altitude (>1.96 km)	–	0.91 (0.82–1.02)
NHANES	NHANES Obesity Prevalence	1.03 (1.03–1.03)	1.03 (1.03–1.04)
	High Altitude (>1.96 km)	–	0.94 (0.84–1.07)
BRFSS	BRFSS Obesity Prevalence	1.05 (1.04–1.05)	1.05 (1.04–1.06)
	High Altitude (>1.96 km)	–	0.86 (0.77–0.97)
Combined‡	REGARDS	1.08 (1.07–1.09)	1.10 (1.09–1.11)
	BRFSS	1.00 (0.99–1.01)	1.01 (1.00–1.02)
	NHANES	0.99 (0.99–1.00)	0.98 (0.97–0.99)
	High Altitude (>1.96 km)	–	0.83 (0.73–0.95)

*Model 1 adjusted for all variables in Tables 1–2 aside from altitude category and housing allowance (branch of service, time in service, age, enlistment BMI, sex, Race/Ethnicity, and job category).

† Model 2 was Model 1 plus housing allowance and altitude category.

‡ Combined refers to a single model including all three measures of civilian obesity prevalence.

Results were similar when stratified by occupation, although aircrew personnel did not necessarily derive additional benefit from high altitude duty assignment (Table 4).

**Table 4:** Hazard of Obesity Diagnosis at High Altitude vs. Low when stratified by Occupation.

Occupation	Hazard Ratio
Mototransport/Armor	0.53 (0.36–0.77)
Communications/Intelligence	0.48 (0.39–0.60)
Healthcare	0.46 (0.30–0.69)
Infantry/Artillery/Combat Engineer	0.60 (0.40–0.89)
Other	0.71 (0.60–0.85)
Repair/engineer	0.63 (0.52–0.76)
Aircrew	1.02 (0.14–7.54)

Adjusted for time in service, age, enlistment BMI, sex, Race/Ethnicity, housing allowance, and job category. Adjusted for branch of service if occupation is in both Army and Air Force.

When replacing altitude and civilian obesity prevalence with a categorical variable for census region, the regional variation in obesity incidence demonstrated increased hazard in the center of the country in the two census regions immediately west of the Mississippi River (West North Central and West South Central – Table 5).

**Table 5:** Regional Variation in Obesity Hazard and Relationship with Civilian Obesity.

Census Region	Hazard Ratio (95% CI)	
	Model 1*	Model 2†
New England‡	0.98 (0.68–1.42)	0.88 (0.62–1.26)
Mid Atlantic	1.27 (1.13–1.44)	1.06 (0.96–1.18)
East North Central	1.62 (1.40–1.88)	1.60 (1.39–1.85)
West North Central	2.33 (2.11–2.56)	2.25 (2.06–2.47)
South Atlantic	1.38 (1.26–1.50)	1.29 (1.21–1.38)
East South Central	Referent	1.20 (1.06–1.37)
West South Central	2.51 (2.30–2.73)	2.32 (2.15–2.50)
Mountain**	1.32 (1.20–1.45)	1.14 (1.05–1.23)
Pacific	1.20 (1.09–1.33)	Referent

*Model 1 adjusted for all variables in Tables 1–2 aside from altitude category (branch of service, time in service, age, enlistment BMI, sex, housing allowance, Race/Ethnicity, and job category).

† Model 2 also adjusted for self-reported current smoking among civilians (as reported in 2011 BRFSS).

‡ New England not used as the referent group as it had the smallest sample size.

**The average altitude of 3 digit zip code areas throughout the Mountain census region varied from 0.44 km to 2.87 km (highest residence of a service member was 2.34 km) and a majority of person time observed in this region was from members living at <1.96 km.

State-level prevalence of self reported current smoking among civilians was inversely associated with an obesity diagnosis among military members. For every 1% increase in smoking prevalence, there was a 4% lower hazard of an obesity diagnosis (HR 0.96, 95% CI 0.95–0.97) in the full model. Adjusting for smoking strengthened the inverse association between altitude and obesity hazard. The HR of obesity at high altitude (>1.96 km) decreased to 0.52 (95% CI 0.47–0.58) as compared to those living <0.98 km. Additionally, there was a dose response pattern with those living between 0.98 km and 1.96 km exhibiting a HR of 0.86 (95% CI 0.82–0.91) for new diagnoses of obesity as compared to those living <0.98 km. Adjusting for smoking prevalence also magnified the relationship between altitude and obesity hazard when modeling altitude as a continuous variable. Prior to adjustment the HR was 0.94 (95% CI 0.91–0.96) and after adjustment it was 0.86 (95% CI 0.83–0.89) per kilometer above sea level.

## Discussion

Among overweight service members in the U.S. Army and Air Force between January 2006 and December 2012, those stationed at higher altitude duty locations had a lower incidence of obesity. This provides the first evidence of a longitudinal association between living at high altitude and long-term obesity protection.

Both behavioral and biological mechanisms could explain this finding. While the association between civilian obesity prevalence and military obesity incidence could reflect shared behavioral mechanisms (e.g., a common built environment), social norming, and social transmission, [50], [51] service members are partially shielded from these mechanisms by having consistent access to healthy foods at military commissaries, a peer group of other military members, and incentives to remain physically active, such as periodic physical fitness tests.

On the contrary, this association could reflect shared biological exposures, such as environmental pollutants, climatic factors, and hypoxia. Although this study cannot entirely discriminate between these exposures, it provides support for the hypoxia hypothesis. First, high altitude duty assignment conferred a significant protective effect even after adjusting for a home of record of Colorado, the state with the lowest obesity prevalence. Second, this association pertained to all occupational strata other than aircrew, who were protected regardless of duty location, reflecting perhaps their intermittent exposure to the hypobaric conditions of aircraft.

The role of hypoxia is also consistent with several hormonal mechanisms proposed by other investigators. Leptin, which helps suppress appetite, is transcribed under the influence of hypoxia inducible factor (HIF) [52]. Some have found serum leptin rises at high altitude, [53] and the leptin *receptor* also appears upregulated by hypoxia. [54] Thus, leptin transcription and signaling alterations could contribute to appetite changes even if serum concentration is unchanged. Likewise, other proposed hormonal mediators such as cholecystokinin (CCK) [28] and norepinethrine have been shown to increase at altitude, [55], [56] which could influence appetite directly (CCK) or indirectly (norepinephrine) via reduced blood flow to the gut [57]. Erythropoietin (EPO), which prevents obesity in mice through non-erythroid receptors, [58] is another potential factor. Although the extent to which endogenous EPO fluctuates with altitude is unclear, it is clearly related to hypoxia and dosing requirements of exogenous EPO in kidney failure patients are reduced at high altitude. [59] EPO phosphylates paroxysome proliferator-activated receptor γ, [58] which is the so-called “master switch” of adipocyte development [60].

While this analysis featured chronic exposure to hypobaric pressure among those stationed at high altitude, future studies could explore whether such conditions are necessary to achieve a similar effect. Long-term exposure to normobaric hypoxemia in chronic lung disease is associated with cachexia, which is reversed with ventilatory support, [61] and short-term pulsatile hypoxia (such as in an altitude tent or pressurized aircraft) has also been proposed as a therapy for obesity. [12], [23] Furthermore, other normobaric alternatives could be investigated. For instance, rodents experimentally administered cyanamide [62] or carbon monoxide [63] have lower body weight. Carbon monoxide, which has been proposed as a therapeutic agent for a range of human diseases, [64] can be delivered in small doses that are nonfatal (such as those provided by secondhand cigarette smoke exposure) [65] stimulated endogenously through HO-1 inducers, [66], [67] or given by other delivery mechanisms. [68] In fact, the HO-1 inducers are already known to stimulate weight loss and prevent obesity in rodents. [66], [67] Furthermore, previously documented connections between ferritin, phlebotomy, and insulin resistance [69], [70] have been shown to connect through the (hypoxia related) HIF1α pathway [71].

When considering hypoxia as a therapeutic agent, the potential risks warrant caution. Obese individuals may be at greater risk for altitude sickness, particularly at an altitude above 3600 meters. [72] This provides additional support for the use of hypoxia as a preventive–as in our study–rather than as a therapeutic agent. Mental illness constitutes another potential harmful association with hypoxia exposure, as demonstrated recently by the frequency of suicide and cocaine abuse at high altitude. [7], [73] Hypophagia, while therapeutic for obesity, is also a symptom of depression. Therefore, even if the metabolic effects of hypoxia are clearly favorable, the holistic balance of benefits and harms may tilt in either direction based on the individual patient.

This study featured a large, relatively homogenous population with longer follow up than any previous study identified. Although the findings are consistent with earlier interventional trials, [20]–[28], [74] its longer duration provides a notable contribution given the limitations of short-term trials. First, since obesity is a chronic disease, short-term benefits are of questionable public health utility unless they are sustainable. Second, humans adapt to high altitude exposure, so any short-term effect could be attributable to the physiologic changes associated with the adaptation process rather than a steady state effect at altitude. Third, some adaptations to high altitude could modify body weight without altering body fat. Hemoconcentration, for example, would reduce total body water and body mass without reducing body fat [75].

This study’s quasi-experimental design–by which the intervention of duty assignment was neither chosen by the participants nor randomly allocated by the investigators–reduced the likelihood of residual confounding (i.e., the outcome of obesity would more likely reflect the impact of residence itself, rather than the participants’ choice of residence). Additionally, the study design is inherently translational due to the real world conditions which allows assessment of effectiveness more than efficacy. [76] Finally, outcomes were determined by healthcare providers in the routine course of patient care who had no knowledge of this investigation.

Nonetheless, the study should be interpreted in light of its limitations. Although service members are not free to reside anywhere in the United States, the assignment process is not entirely random. Those with a healthier lifestyle may have been more likely to request assignment at high altitude, although data on such requests are not available. The homogeneity of effect across all career fields, however, suggests the assignment process is not responsible for the association seen. Another limitation is the use of the so-called “permanent” duty location to define exposure. Unplanned cross-over likely occurred during military leave, deployments, and temporary duty, thus biasing our results toward the null. In addition, potential confounding variables (e.g., smoking status) were not measured. Adjustment for civilian smoking prevalence suggested this missing data biased our results to the null. This adjustment also suggested that missing smoking data may have partially explained the lower than expected obesity hazard in the East South Central census region (Table 5).

While this study’s military population presents a unique opportunity to evaluate a large cohort of frequently migrating humans, it could be argued that such findings are not generalizable to the civilian population. Several features of this study, however, favor a broader applicability of its findings beyond the U.S. military. First, it evaluates an association previously documented among civilians [32] using a quasi-experimental design. Second, the findings suggest that service members in the study mirrored geographically collocated civilians as it relates to obesity outcomes. In fact, incident obesity diagnoses occurred at rates proportionate to the prevalence of obesity in the local civilian population. This finding is not surprising since the military is made up of a socioeconomically diverse source population of civilians who previously resided throughout the United States. Third, although screening and training of applicants results in a healthy working population, this is not dissimilar to the recruitment process of clinical trials featuring healthy subjects. Fourth, military personnel in this study were only included after completing at least two years of service, and thus had moved beyond training settings with mandated dietary choices and physical training programs. Finally, results were consistent across occupational substrata. If the effect of altitude on obesity were contingent on something unique to the military lifestyle, one would expect occupational substrata classically associated with a military ethos (e.g., infantry) to demonstrate a stronger effect size than those in other fields, such as healthcare.

Aside from the translational findings relating to obesity, our study also provides at least two methodological contributions for making causal inferences of spatial determinism. Although the classic components of descriptive epidemiology (i.e., person, place, and time) demonstrate the importance of place in disease pattern recognition, analytic techniques are needed to evaluate if a disease is merely located in a particular place, or if that place actually exerts a causal influence on the development of the disease. Our first contribution to this end is the identification of the U.S. military as an ideal source population for investigating the causal influence of geographic locations on health. In addition to the characteristics we have outlined (i.e., frequent and assigned migration, data availability, and generalizability), this population is also appropriate because military members may benefit from knowledge of the unique health risks and rewards associated with moving to new places. We also demonstrated a novel method of etiologic assessment using external data. We compared disease prevalence among the extant local population with disease incidence among residents who had recently migrated there, thus accounting for unmeasured factors. In this case, by finding an association between civilian obesity prevalence and military obesity incidence even in low altitude areas, we suspect that geographic determinants of obesity likely extend beyond altitude alone. Similarly, we identified and adjusted for one spatial factor (variance in smoking behaviors) that could impact newly arrived residents differently than long term residents.

In summary, high altitude duty assignment of overweight U.S. military service members is associated with lower rate of obesity diagnoses as compared with low altitude duty assignment, even after adjusting for state level obesity prevalence. Census region residence is a newly identified modifiable risk factor for obesity for this population. Furthermore, the study raises many new avenues of research with significant implications for global health.
